# Supplementary material for: The Systems Biology Research Tool: evolvable open-source software
Source: BMC Syst Biol. 2008 Jun 29;2:55. doi: 10.1186/1752-0509-2-55 (PMC2446383; doi:10.1186/1752-0509-2-55)
Supplement: Additional file 1 — SBRT Archive. An archive of the current version of the Systems Biology Research Tool. [file 1752-0509-2-55-S1.zip › sbrt-1.4.0/doc/developers_guide/api/index-files/index-9.html]

I-Index


|  |  |  |
| --- | --- | --- |
| **Overview** \| Package \| Class \| Use \| **Tree** \| **Deprecated** \| **Index** \| **Help** | |  |
| **PREV LETTER**   **NEXT LETTER** | **FRAMES**    **NO FRAMES**     **All Classes** |  |


A B C D E F G H I J K L M N O P Q R S T U V W X Y Z 

---


## **I**

**IllegalBoundsException** - Exception in sbrt.kernel.math: This class is used to indicate the existence of illegal bounds. **IllegalBoundsException(Number, Number)** - Constructor for exception sbrt.kernel.math.IllegalBoundsException: Constructs a new illegal bounds exception from the provided lower and upper bounds. **IllegalBoundsException(double, double)** - Constructor for exception sbrt.kernel.math.IllegalBoundsException: Constructs a new illegal bounds exception from the provided lower and upper bounds. **IllegalBoundsException(Object, double, double)** - Constructor for exception sbrt.kernel.math.IllegalBoundsException: Constructs a new illegal bounds exception from the provided lower and upper bounds. **IllegalBoundsException(Object, Number, Number)** - Constructor for exception sbrt.kernel.math.IllegalBoundsException: Constructs a new illegal bounds exception from the provided lower and upper bounds. **IllegalFreeVariableException** - Exception in sbrt.kernel.math.algebra: This class is used to indicate the presence of an illegal free variable. **IllegalFreeVariableException(Object)** - Constructor for exception sbrt.kernel.math.algebra.IllegalFreeVariableException: Constructs a new illegal free variable exception. **inactive()** - Method in class sbrt.kernel.fba.proc.FluxActivityProcess.Results: Returns the number of inactive fluxes. **INACTIVE** - Static variable in class sbrt.shell.mng.fba.RandomConstraintsManager: The keyword used to denote the inactive constraint. **increment()** - Method in class sbrt.kernel.util.AbstractProgressMonitor: Causes this progress monitor to increment an internal counter. **increment()** - Method in interface sbrt.kernel.util.MutableProgressMonitor: Causes this progress monitor to increment an internal counter. **increment()** - Method in class sbrt.kernel.util.PercentProgressMonitor: Causes this progress monitor to increment an internal counter. **indexOf(Object)** - Method in class sbrt.kernel.math.graph\_theory.Path: Returns the index in this path of the first occurrence of the specified node, or -1 if this path does not contain that node. **indexOf(Object)** - Method in class sbrt.kernel.util.UnmodifiableList: See List.indexOf(Object). **IndexPair**<R,C> - Class in sbrt.kernel.math.algebra: This class is used to represent pairs of matrix indices. **IndexPair(S, T)** - Constructor for class sbrt.kernel.math.algebra.IndexPair: Constructs a new pair of matrix indices. **IndexPairFormat**<R,C> - Interface in sbrt.shell.text.math: This interface is used to represent formats for pairs of indices. **IndexPairFormatV1**<R,C> - Class in sbrt.shell.text.math: This class is a concrete implementation of `IndexPairFormat`. **IndexPairFormatV1(SimpleFormat<R>, SimpleFormat<C>)** - Constructor for class sbrt.shell.text.math.IndexPairFormatV1: Constructs a new index pair format. **InfeasibleSolutionException** - Exception in sbrt.kernel.math: This class is used to indicate that no feasible solution exists. **InfeasibleSolutionException(Object)** - Constructor for exception sbrt.kernel.math.InfeasibleSolutionException: Constructs a new infeasible solution exception. **InfeasibleSolutionException(Object, String)** - Constructor for exception sbrt.kernel.math.InfeasibleSolutionException: Constructs a new infeasible solution exception. **InfeasibleSolutionException(Object, Throwable)** - Constructor for exception sbrt.kernel.math.InfeasibleSolutionException: Constructs a new infeasible solution exception. **InfeasibleSolutionException(Object, String, Throwable)** - Constructor for exception sbrt.kernel.math.InfeasibleSolutionException: Constructs a new infeasible solution exception. **INITIAL\_POINT\_FILE** - Static variable in class sbrt.shell.mng.geom.CdHarManager: The keyword used to denote the initial point file. **InitialPointComputer** - Class in sbrt.kernel.fba.comp: This class is used to compute initial points for coordinate-direction hit-and-run random flux vector algorithms. **InitialPointComputer(FbaOptComputer)** - Constructor for class sbrt.kernel.fba.comp.InitialPointComputer: Constructs a new initial point, or flux vector, computer. **InitialPointComputer.Results** - Class in sbrt.kernel.fba.comp: This class is used to represent the results of a **InitialPointGenerator** - Class in sbrt.kernel.fba.proc: This class is used to perform initial point experiments. **InitialPointGenerator(InitialPointComputer)** - Constructor for class sbrt.kernel.fba.proc.InitialPointGenerator: Constructs a new initial point experiment. **InitialPointManager** - Class in sbrt.shell.mng.fba: This class is used to manage initial point experiments. **InitialPointManager()** - Constructor for class sbrt.shell.mng.fba.InitialPointManager: **INPUT\_FILE** - Static variable in class sbrt.shell.mng.AbstractProcessManager: The keyword used to denote the name of the input file. **INPUT\_FILE\_FORMAT** - Static variable in class sbrt.shell.mng.AbstractProcessManager: The keyword used to denote the name of an input file format. **INPUT\_FILE\_NAME\_FILE** - Static variable in class sbrt.shell.mng.AbstractProcessManager: The keyword used to denote the name of a file containing a list of file names. **INPUT\_RXN\_FILE** - Static variable in class sbrt.shell.mng.fba.SimpleRxnFileTransManager: The keyword used to denote the name of the input reaction file. **InputFile**<E> - Interface in sbrt.shell.io: This interface is used to represent input files. **IntegerFormat** - Interface in sbrt.shell.text: This interface is used to represent a format for integers. **IntegerFormatV1** - Class in sbrt.shell.text: This class is a concrete implementation of `IntegerFormat`. **IntegerFormatV1()** - Constructor for class sbrt.shell.text.IntegerFormatV1: **internalRxns()** - Method in interface sbrt.kernel.fba.Fluxome: Returns the total number of internal reactions contained in this fluxome. **internalRxns()** - Method in class sbrt.kernel.fba.IrrevRxnFluxomeV1: Returns the total number of internal reactions contained in this fluxome. **interpret(Throwable)** - Method in class sbrt.ProcessRunner: Interprets the provided throwable. **interpret(ExternalSoftwareException)** - Static method in class sbrt.shell.mng.algebra.LinearSolverManager: Interprets the provided external software exception. **interpret(RuntimeException)** - Static method in class sbrt.shell.mng.fba.CdHarFluxVectorManager: Attempts to interpret the provided RuntimeException. **interpret(DimensionException)** - Static method in class sbrt.shell.mng.fba.CdHarFluxVectorManager: Returns an `ApplicationException` whose message describes the error that ocurred. **interpret(InfeasibleSolutionException)** - Static method in class sbrt.shell.mng.fba.CdHarFluxVectorManager: Returns an `ApplicationException` whose message describes the error that ocurred. **interpret(UnboundedVariableException)** - Static method in class sbrt.shell.mng.fba.CdHarFluxVectorManager: Returns an `ApplicationException` whose message describes the error that ocurred. **interpret(InvalidFluxVectorException)** - Static method in class sbrt.shell.mng.fba.CdHarFluxVectorManager: Returns an `ApplicationException` whose message describes the error that ocurred. **interpret(ExceededLimitException)** - Static method in class sbrt.shell.mng.fba.CdHarFluxVectorManager: Returns an `ApplicationException` whose message describes the error that ocurred. **interpret(RuntimeException, FbaProcess<?>)** - Static method in class sbrt.shell.mng.fba.FbaOptManager: Returns an `ApplicationException` whose message describes the error that ocurred. **interpret(ProgramSolverException)** - Static method in class sbrt.shell.mng.fba.FbaOptManager: Returns an `ApplicationException` whose message describes the error that ocurred. **interpret(ConstraintViolationException, FbaProcess<?>)** - Static method in class sbrt.shell.mng.fba.FbaOptManager: Returns an `ApplicationException` whose message describes the error that ocurred. **interpret(FbaOptException, FbaProcess<?>)** - Static method in class sbrt.shell.mng.fba.FbaOptManager: Returns an `ApplicationException` whose message describes the error that ocurred. **interpret(FluxRangeException, FbaProcess)** - Static method in class sbrt.shell.mng.fba.MsFluxIntervalManager: Returns an `ApplicationException` whose message describes the error that ocurred. **interpret(IllegalBoundsException)** - Static method in class sbrt.shell.mng.fba.SimpleFluxIntervalManager: Returns an `ApplicationException` whose message describes the error that ocurred. **interpret(UnboundedVariableException)** - Static method in class sbrt.shell.mng.geom.CdHarManager: Returns an `ApplicationException` whose message describes the error that ocurred. **interpret(ExceededLimitException)** - Static method in class sbrt.shell.mng.geom.CdHarManager: Returns an `ApplicationException` whose message describes the error that ocurred. **interpret(JriException)** - Static method in class sbrt.shell.mng.stat.CorrelationManager: Wraps the provided JRI exception as an application exception. **interpret(ProgramSolverException)** - Static method in class sbrt.shell.ProgramSolverFactory: Interprets a program solver exception and returns a corresponding application exception. **interpretSolution(Expr)** - Static method in class sbrt.ext.mma.algebra.MMaExprInterpretter: Converts the provided Mathematica expression to a `Map`. **Interval** - Class in sbrt.kernel.math: This class is used to represent closed intervals of the form [α, β], where α ≤ β. **Interval(double, double)** - Constructor for class sbrt.kernel.math.Interval: Constructs a new interval with the provided lower and upper bounds. **IntervalComparer** - Class in sbrt.kernel.math: This class is used to compare vectors of intervals. **IntervalComparer()** - Constructor for class sbrt.kernel.math.IntervalComparer: **IntervalComparer.Diff**<V> - Class in sbrt.kernel.math: Instances of this class are used to hold a variable and a pair of corresponding intervals. **IntervalComparison**<V> - Class in sbrt.kernel.utilities.proc: This class is used to compare sets of intervals. **IntervalComparison()** - Constructor for class sbrt.kernel.utilities.proc.IntervalComparison: **IntervalComparisonManager** - Class in sbrt.shell.mng.utilities: This class is used to compare the intervals contained in separate files. **IntervalComparisonManager()** - Constructor for class sbrt.shell.mng.utilities.IntervalComparisonManager: **IntervalEquiv** - Class in sbrt.kernel.math: This class is used to compare intervals for equivalence. **IntervalEquiv(double)** - Constructor for class sbrt.kernel.math.IntervalEquiv: Creates a new interval equivalence tester with the provided tolerance. **IntervalFormat** - Interface in sbrt.shell.text.math: This interface is used to represent formats for closed intervals. **IntervalFormatV1** - Class in sbrt.shell.text.math: This class is a concrete implementation of `IntervalFormat`. **IntervalFormatV1()** - Constructor for class sbrt.shell.text.math.IntervalFormatV1: Constructs a new interval format. **IntervalVariationManager** - Class in sbrt.shell.mng.fba: This abstract class provides utitlity functions for managing flux interval variation experiments. **IntervalVariationManager()** - Constructor for class sbrt.shell.mng.fba.IntervalVariationManager: **InvalidFluxVectorException** - Exception in sbrt.kernel.fba.comp: This class is used to indicate that a flux vector is invalid. **InvalidFluxVectorException(Map<String, Double>)** - Constructor for exception sbrt.kernel.fba.comp.InvalidFluxVectorException: Constructs a new exception for the provided flux vector. **InvalidFluxVectorException(Map<String, Double>, String)** - Constructor for exception sbrt.kernel.fba.comp.InvalidFluxVectorException: Constructs a new exception for the provided flux vector. **InvalidFluxVectorException(Map<String, Double>, Throwable)** - Constructor for exception sbrt.kernel.fba.comp.InvalidFluxVectorException: Constructs a new exception for the provided flux vector. **InvalidFluxVectorException(Map<String, Double>, String, Throwable)** - Constructor for exception sbrt.kernel.fba.comp.InvalidFluxVectorException: Constructs a new exception for the provided flux vector. **InvalidPointException** - Exception in sbrt.kernel.math: This class is used to indicate that a point is invalid. **InvalidPointException(Map<?, ?>)** - Constructor for exception sbrt.kernel.math.InvalidPointException: Constructs an exception to indicate the provided point is invalid. **InvalidPointException(Map<?, ?>, String)** - Constructor for exception sbrt.kernel.math.InvalidPointException: Constructs an exception to indicate the provided point is invalid. **IrfFormatV1** - Class in sbrt.shell.text.fba: This class is used to format irreversible reaction fluxome files. **IrfFormatV1()** - Constructor for class sbrt.shell.text.fba.IrfFormatV1: Constructs a new irreversible reaction fluxome format. **IrrevRxn** - Class in sbrt.kernel.fba: This class is used to represent irreversible chemical reactions. **IrrevRxn(LinearComb<String>, LinearComb<String>)** - Constructor for class sbrt.kernel.fba.IrrevRxn: Constructs a new irreversible reaction from the provided reactants and products. **IrrevRxnFluxome** - Interface in sbrt.kernel.fba: This class is used to represent fluxomes that contain only irreversible reactions. **IrrevRxnFluxomeV1** - Class in sbrt.kernel.fba: This class is a concrete implementation of IrrevRxnFluxome. **IrrevRxnFluxomeV1(Map<String, IrrevRxn>, Map<String, SourceRxn>, Map<String, SinkRxn>, Map<String, Or<And<String>>>)** - Constructor for class sbrt.kernel.fba.IrrevRxnFluxomeV1: Constructs a new irreversible reaction fluxome from the provided internal and exchange reactions and catalysts. **IrrevRxnFluxomeV1(Map<String, IrrevRxn>, Map<String, SourceRxn>, Map<String, SinkRxn>)** - Constructor for class sbrt.kernel.fba.IrrevRxnFluxomeV1: Constructs a new irreversible reaction fluxome from the provided internal and exchange reactions. **IrrevRxnFluxomeV1(Map<String, ChemRxn>, Map<String, Or<And<String>>>)** - Constructor for class sbrt.kernel.fba.IrrevRxnFluxomeV1: Constructs a new irreversible reaction fluxome from the provided internal and exchange reactions and catalysts. **IrrevRxnFluxomeV1(Map<String, ChemRxn>)** - Constructor for class sbrt.kernel.fba.IrrevRxnFluxomeV1: Constructs a new irreversible reaction fluxome from the provided internal and exchange reactions. **IrrevRxnFormatV1** - Class in sbrt.shell.text.chem: This class is used to format irreversible chemical reactions. **IrrevRxnFormatV1()** - Constructor for class sbrt.shell.text.chem.IrrevRxnFormatV1: Constructs a new irreversible reaction format. **IrrevRxnFormatV1** - Class in sbrt.shell.text.fba: This class is used to format irreversible reactions for use in flux balance analysis. **IrrevRxnFormatV1()** - Constructor for class sbrt.shell.text.fba.IrrevRxnFormatV1: Constructs a new irreversible reaction format. **IrrevRxnFormatV2** - Class in sbrt.shell.text.chem: This class is used to format irreversible chemical reactions. **IrrevRxnFormatV2()** - Constructor for class sbrt.shell.text.chem.IrrevRxnFormatV2: Constructs a new irreversible reaction format. **isCatalyst(String)** - Method in interface sbrt.kernel.fba.CatalyzedFluxome: Indicates if the provided string is the name of a catalyst contained in this fluxome. **isCatalyst(String)** - Method in class sbrt.kernel.fba.IrrevRxnFluxomeV1: Indicates if the provided string is the name of an catalyst contained in this fluxome. **isClosed()** - Method in class sbrt.kernel.io.AbstractResultsManager: Indicates if this results manager has already been closed. **isClosed()** - Method in interface sbrt.kernel.io.ResultsManager: Indicates if this results manager has already been closed. **isClosed()** - Method in class sbrt.shell.io.AbstractMapInputFile: Indicates if this file has already been closed. **isClosed()** - Method in class sbrt.shell.io.AbstractMapOutputFile: Indicates if this file has already been closed. **isClosed()** - Method in class sbrt.shell.io.fba.ColumnOrFbaOptOutputFile: Indicates if this file has already been closed. **isClosed()** - Method in class sbrt.shell.io.fba.FluxCapInputFile: Indicates if this file has already been closed. **isClosed()** - Method in class sbrt.shell.io.fba.FluxIntervalInputFile: Indicates if this file has already been closed. **isClosed()** - Method in class sbrt.shell.io.fba.RowOrFbaOptOutputFile: Indicates if this file has already been closed. **isClosed()** - Method in class sbrt.shell.io.fba.SerFbaOptOutputFile: Indicates if this file has already been closed. **isClosed()** - Method in class sbrt.shell.io.MultiOutputFiles: Indicates if this results manager has already been closed. **isClosed()** - Method in interface sbrt.shell.io.SbrtFile: Indicates if this file has already been closed. **isClosed()** - Method in class sbrt.shell.io.SerializedFileIterator: Indicates if this iterator has been closed. **isClosed()** - Method in class sbrt.shell.io.SerializedInputFile: Indicates if this file has already been closed. **isClosed()** - Method in class sbrt.shell.io.SerializedOutputFile: Indicates if this file has already been closed. **isClosed()** - Method in class sbrt.shell.io.TextFileLineIterator: Indicates if this iterator has been closed. **isClosed()** - Method in class sbrt.shell.io.TextInputFile: Indicates if this file has already been closed. **isClosed()** - Method in class sbrt.shell.io.TextOutputFile: Indicates if this file has already been closed. **isColumnIndex(Object)** - Method in class sbrt.kernel.math.algebra.HashMatrix: Indicates if the provided object is a column index in this matrix. **isColumnIndex(Object)** - Method in interface sbrt.kernel.math.algebra.Matrix: Indicates if the provided object is a column index in this matrix. **isColumnIndex(Object)** - Method in class sbrt.kernel.math.algebra.MatrixWrap: Indicates if the provided object is a column index in this matrix. **isColumnIndex(Object)** - Method in class sbrt.kernel.math.algebra.RowOrTreeMatrix: Indicates if the provided object is a column index in this matrix. **isColumnIndex(Object)** - Method in class sbrt.kernel.math.algebra.TreeMatrix: Indicates if the provided object is a column index in this matrix. **isColumnVar(Object)** - Method in class sbrt.ext.cplex.CplexSolver: Indicates if the provided object is a column variable in the matrix of this program solver. **isColumnVar(Object)** - Method in class sbrt.ext.glpk.GlpkSolver: Indicates if the provided object is a column variable in the matrix of this program solver. **isColumnVar(Object)** - Method in interface sbrt.kernel.math.optimization.ProgramSolver: Indicates if the provided object is a column variable in the matrix of this program solver. **isEdge(Object, Object)** - Method in class sbrt.kernel.math.graph\_theory.DirectedGraph: Indicates if an edge exists between the provided nodes. **isEmpty()** - Method in class sbrt.kernel.math.algebra.AbstractMdMatrix: Indicates if this matrix is empty. **isEmpty()** - Method in interface sbrt.kernel.math.algebra.Matrix: Indicates if this matrix is empty. **isEmpty()** - Method in class sbrt.kernel.math.algebra.MatrixWrap: Indicates if this matrix is empty. **isEmpty()** - Method in class sbrt.kernel.util.UnmodifiableCollection: See Collection.isEmpty(). **isEmpty()** - Method in class sbrt.kernel.util.UnmodifiableList: See List.isEmpty(). **isEmpty()** - Method in class sbrt.kernel.util.UnmodifiableMap: See Map.isEmpty(). **isEmpty()** - Method in class sbrt.kernel.util.UnmodifiableSet: See Set.isEmpty(). **isEof(Object)** - Static method in class sbrt.shell.io.EndOfFile: Indicates if the provided object is an instance of this class. **isEof(Object)** - Static method in class sbrt.shell.io.SerializedOutputFile: Indicates if the provided object is a valid end-of-file marker. **isEquivalent(ChemRxn)** - Method in interface sbrt.kernel.fba.ChemRxn: Indicates if this chemical reaction is stoichiometrically equivalent to the provided reaction. **isEquivalent(ChemRxn)** - Method in class sbrt.kernel.fba.IrrevRxn: Indicates if the provided chemical reaction is stoichiometrically equivalent to this reaction. **isEquivalent(ChemRxn)** - Method in class sbrt.kernel.fba.ReversibleRxn: Indicates if the provided chemical reaction is stoichiometrically equivalent to this reaction. **isEquivalent(ChemRxn)** - Method in class sbrt.kernel.fba.SinkRxn: Indicates if the provided chemical reaction is stoichiometrically equivalent to this sink reaction. **isEquivalent(ChemRxn)** - Method in class sbrt.kernel.fba.SourceRxn: Indicates if the provided chemical reaction is stiochiometrically equivalent to this source reaction. **isFluxVector(Map<String, Double>, double)** - Method in interface sbrt.kernel.fba.Fluxome: Indicates if the provided flux vector lies within the feasible space of this fluxome, given the provided tolerance value. **isFluxVector(Map<String, Double>, double)** - Method in class sbrt.kernel.fba.IrrevRxnFluxomeV1: Indicates if the provided flux vector lies within the feasible space of this fluxome, given the provided tolerance value. **isKey(Object)** - Static method in class sbrt.ext.R.R: Indicates if the provided object is the key to unlock the R engine. **isLocked()** - Static method in class sbrt.ext.R.R: Indicates if the R engine is currently locked. **isNode(Object)** - Method in class sbrt.kernel.math.graph\_theory.DirectedGraph: Indicates if the provided object is a node in this graph. **isObjValueHeader(String)** - Method in interface sbrt.shell.text.fba.FbaOptFileLineFormat: Indicates if the provided string is the header used to indicate the value of the objective function. **isObjValueHeader(String)** - Method in class sbrt.shell.text.fba.FbaOptFileLineFormatV1: Indicates if the provided string is the header used to indicate the value of the objective function. **isObjValueHeader(String)** - Method in interface sbrt.shell.text.fba.FbaOptHeaderFormatter: Indicates if the provided string is the header used to indicate the value of the objective function. **isObjValueHeader(String)** - Method in class sbrt.shell.text.fba.FbaOptHeaderFormatterV1: Indicates if the provided string is the header used to indicate the value of the objective function. **isObjValueHeader(String)** - Method in interface sbrt.shell.text.fba.FbaOptHeaderParser: Indicates if the provided string is the header used to indicate the value of the objective function. **isObjValueHeader(String)** - Method in class sbrt.shell.text.fba.FbaOptHeaderParserV1: Indicates if the provided string is the header used to indicate the value of the objective function. **isReqCatalyst(String, String)** - Method in interface sbrt.kernel.fba.CatalyzedFluxome: Indicates if the provided catalyst is required for the reaction with the provided name. **isReqCatalyst(String, String)** - Method in class sbrt.kernel.fba.IrrevRxnFluxomeV1: Indicates if the specified catalyst is required for the reaction with the specified name. **isReverse(ChemRxn)** - Method in interface sbrt.kernel.fba.ChemRxn: Indicates if the provided chemical reaction is the reverse of this reaction. **isReverse(ChemRxn)** - Method in class sbrt.kernel.fba.IrrevRxn: Indicates if the provided chemical reaction is the reverse of this reaction. **isReverse(ChemRxn)** - Method in class sbrt.kernel.fba.ReversibleRxn: Returns `false`, since no reaction can be the "reverse" of a reversible reaction. **isReverse(ChemRxn)** - Method in class sbrt.kernel.fba.SinkRxn: Indicates if the provided chemical reaction is the reverse of this sink reaction. **isReverse(ChemRxn)** - Method in class sbrt.kernel.fba.SourceRxn: Indicates if the provided chemical reaction is the reverse of this source reaction. **isReversible(Node)** - Method in class sbrt.shell.text.fba.BiggRxnNodeParser: Indicates if the reaction in the provided reaction node is reversible. **isReversible(Node)** - Method in class sbrt.shell.text.fba.PalssonRxnNodeParser: Indicates if the reaction in the provided reaction node is reversible. **isRowIndex(Object)** - Method in class sbrt.kernel.math.algebra.HashMatrix: Indicates if the provided object is a row index in this matrix. **isRowIndex(Object)** - Method in interface sbrt.kernel.math.algebra.Matrix: Indicates if the provided object is a row index in this matrix. **isRowIndex(Object)** - Method in class sbrt.kernel.math.algebra.MatrixWrap: Indicates if the provided object is a row index in this matrix. **isRowIndex(Object)** - Method in class sbrt.kernel.math.algebra.RowOrTreeMatrix: Indicates if the provided object is a row index in this matrix. **isRowIndex(Object)** - Method in class sbrt.kernel.math.algebra.TreeMatrix: Indicates if the provided object is a row index in this matrix. **isRowVar(Object)** - Method in class sbrt.ext.cplex.CplexSolver: Indicates if the provided object is a row variable in the matrix of this program solver. **isRowVar(Object)** - Method in class sbrt.ext.glpk.GlpkSolver: Indicates if the provided object is a row variable in the matrix of this program solver. **isRowVar(Object)** - Method in interface sbrt.kernel.math.optimization.ProgramSolver: Indicates if the provided object is a row variable in the matrix of this program solver. **isRxnName(String)** - Method in interface sbrt.kernel.fba.Fluxome: Indicates if the provided string is the name of a reaction contained in this fluxome. **isRxnName(String)** - Method in class sbrt.kernel.fba.IrrevRxnFluxomeV1: Indicates if the provided string is the name of a reaction contained in this fluxome. **isSolutionVector(Map<?, Double>, double)** - Method in class sbrt.kernel.math.algebra.HashLinearSystemSolution: Indicates if the provided solution vector is consistent with this solution. **isSolutionVector(Map<?, Double>, double)** - Method in interface sbrt.kernel.math.algebra.LinearSystemSolution: Indicates if the provided solution vector is consistent with this solution. **isSpecies(String)** - Method in interface sbrt.kernel.fba.Fluxome: Indicates if the provided string is the name of a chemical species contained in this fluxome. **isSpecies(String)** - Method in class sbrt.kernel.fba.IrrevRxnFluxomeV1: Indicates if the provided string is the name of a chemical species contained in this fluxome. **isValid(Interval, String, double)** - Method in interface sbrt.kernel.fba.Fluxome: Indicates if the provided interval is a valid flux constraint for the reaction with the provided name. **isValid(Interval, String, double)** - Method in class sbrt.kernel.fba.IrrevRxnFluxomeV1: Indicates if the provided interval is a valid flux constraint for the reaction having the provided name. **isValidFormat(FileFormat)** - Static method in class sbrt.shell.io.SerializedFileIterator: Indicates if the provided file format is valid for this type of file iterator. **isValidFormat(FileFormat)** - Static method in class sbrt.shell.io.SerializedInputFile: Indicates if the provided file format is valid for a text input file. **isValidFormat(FileFormat)** - Static method in class sbrt.shell.io.SerializedOutputFile: Indicates if the provided file format is valid for a serialized output file. **isValidFormat(FileFormat)** - Static method in class sbrt.shell.io.TextFileLineIterator: Indicates if the provided file format is valid for this type of file line iterator. **isValidFormat(FileFormat)** - Static method in class sbrt.shell.io.TextInputFile: Indicates if the provided file format is valid for a text input file. **isValidFormat(FileFormat)** - Static method in class sbrt.shell.io.TextOutputFile: Indicates if the provided file format is valid for a text output file. **isVariable(Object)** - Method in class sbrt.kernel.math.algebra.HashLinearSystemSolution: Indicates if the provided object is a variable in this solution. **isVariable(Object)** - Method in interface sbrt.kernel.math.algebra.LinearSystemSolution: Indicates if the provided object is a variable in this solution. **isVariable(Object)** - Method in interface sbrt.kernel.math.geom.ConvexPolytope: Indicates if the provided object is a variable in the bounding hyperplanes of this convex polytope. **isVariable(Object)** - Method in class sbrt.kernel.math.geom.HashConvexPolytope: Indicates if the provided object is a variable in the bounding hyperplanes of this convex polytope. **isVariable(Object)** - Method in class sbrt.kernel.math.geom.HashConvexPolytopeOld: Indicates if the provided object is a variable in the bounding hyperplanes of this convex polytope. **isWithin(Map<? extends V, Double>, double)** - Method in interface sbrt.kernel.math.geom.ConvexPolytope: Indicates if the provided point lies within this convex polytope. **isWithin(Map<? extends V, Double>, double)** - Method in class sbrt.kernel.math.geom.HashConvexPolytope: Indicates if the provided point lies within this convex polytope. **isWithin(Map<? extends V, Double>, double)** - Method in class sbrt.kernel.math.geom.HashConvexPolytopeOld: Indicates if the provided point lies within this convex polytope. **isWithin(double)** - Method in class sbrt.kernel.math.Interval: Indicates if the provided value lies within this interval. **isWithin(double, double)** - Method in class sbrt.kernel.math.Interval: Indicates if the provided value lies within this interval, given the provided numerical tolerance. **isZero(double)** - Static method in class sbrt.kernel.util.Util: Indicates if the provided value equals 0. **ITERATIONS** - Static variable in class sbrt.shell.mng.AbstractProcessManager: The keyword used to denote the number of iterations. **iterator()** - Method in class sbrt.kernel.io.ListResultsManager: Returns an iterator over the list of results. **iterator()** - Method in class sbrt.kernel.math.AbstractLinearComb: Returns an iterator over the terms of this linear combination. **iterator()** - Method in class sbrt.kernel.math.AbstractLinearComb.Term: Returns an iterator over the variables in this term. **iterator()** - Method in class sbrt.kernel.math.graph\_theory.Path: Returns an iterator over the nodes in this path in proper sequence. **iterator()** - Method in class sbrt.kernel.math.HashLinearComb: Returns an iterator over the terms of this linear combination. **iterator()** - Method in interface sbrt.kernel.math.LinearComb: Returns an iterator over the terms of this linear combination. **iterator()** - Method in interface sbrt.kernel.math.Polynomial.Term: Returns an iterator over the variables in this term. **iterator()** - Method in class sbrt.kernel.util.BooleanRelation: **iterator()** - Method in class sbrt.kernel.util.UnmodifiableCollection: See Collection.iterator(). **iterator()** - Method in class sbrt.kernel.util.UnmodifiableList: See List.iterator(). **iterator()** - Method in class sbrt.kernel.util.UnmodifiableSet: See Set.iterator(). **iterator()** - Method in class sbrt.shell.io.ComiFile: Returns an iterator over this file. **iterator(int)** - Method in class sbrt.shell.io.ComiFile: Returns an iterator over this file beginning at the specified index. **iterator()** - Method in class sbrt.shell.io.fba.FluxCapInputFile: Returns an iterator over this file. **iterator()** - Method in class sbrt.shell.io.fba.FluxIntervalInputFile: Returns an iterator over the flux intervals contained in this file. **iterator()** - Method in class sbrt.shell.io.fba.RxnNamExprSetInputFile: Returns an iterator over the set of reaction name expressions contained in this file. **iterator()** - Method in class sbrt.shell.io.RomiFile: Returns an iterator over this file. **iterator()** - Method in class sbrt.shell.io.SerializedInputFile: Returns an object iterator over this file. **iterator()** - Method in class sbrt.shell.io.SerializedMapInputFile: Returns an iterator over this file. **iterator()** - Method in class sbrt.shell.io.TextInputFile: Returns a line iterator over this text file. **iterator(int)** - Method in class sbrt.shell.io.TextInputFile: Returns a line iterator over this text file beginning at the specified line.

---


|  |  |  |
| --- | --- | --- |
| **Overview** \| Package \| Class \| Use \| **Tree** \| **Deprecated** \| **Index** \| **Help** | |  |
| **PREV LETTER**   **NEXT LETTER** | **FRAMES**    **NO FRAMES**     **All Classes** |  |


A B C D E F G H I J K L M N O P Q R S T U V W X Y Z 

---
